# Behavioural and Hormonal Stress Responses to Social Separation in Ravens, *Corvus corax*

**DOI:** 10.1111/eth.12580

**Published:** 2016-12-28

**Authors:** Alexandru M. Munteanu, Martina Stocker, Mareike Stöwe, Jorg J. M. Massen, Thomas Bugnyar

**Affiliations:** ^1^ Department of Cognitive BiologyUniversity of ViennaViennaAustria; ^2^ Haidlhof Research StationUniversity of Vienna and University of Veterinary Medicine ViennaBad VöslauAustria; ^3^ Department of Biomedical SciencesUnit of Physiology, Pathophysiology and Experimental EndocrinologyUniversity of Veterinary Medicine ViennaViennaAustria

**Keywords:** social separation, stress, hand‐raising, corticosterone metabolites, coping pattern

## Abstract

Social life is profitable, but it facilitates conflicts over resources and creates interdependence between individuals. Separating highly social animals triggers intense reactions aimed at re‐establishing lost connections. Less is known, however, about behavioural and physiological responses to separation in socially facultative species, where individuals temporarily form groups and may subsequently leave them. Non‐breeding common ravens (*Corvus corax*) gather in large numbers at feeding and roosting sites, but otherwise spend time seemingly solitary or in small subgroups. We here studied how ravens cope with being socially isolated, and investigated the life characteristics that might explain potential individual differences. For this, we individually separated captive subadult ravens (n = 25) and housed them in physical and visual isolation from their group members across 4 d. During the separation period, we collected behavioural data and measured the amount of immunoreactive corticosterone metabolites from bird droppings to assess the ravens’ physiological stress response. We found behavioural indicators of stress at the start of the separation, when ravens showed higher levels of tension than of comfort – a pattern that reversed at the end of the separation. Furthermore, we found that the upbringing of ravens affected their behaviour during separation. Hand‐raised birds produced more vocalisations in the beginning of the separation, and were less active at the end, while the reverse pattern occurred with parent‐raised ravens. Contrary to our predictions, we did not find differences in hormonal responses between the beginning and end of the separation period or any link between hormonal responses and behaviours. Ravens’ behavioural responses to social separation stress seem to be dependent on their arousal states, although possible links with hormonal reactions remain unclear. Our results show that behavioural reactions are not always linked with hormonal responses to stress, and further emphasise the importance of investigating effects of early‐life experiences.

## Introduction

Social life provides animals with many advantages, such as superior protection against predators, more opportunities to acquire information and improved access to resources (Beauchamp 1999, 2014; Galef & Giraldeau 2001; Krause & Ruxton 2002). But group living also comes at a price, as it can promote competition over resources and may facilitate conflicts (Beauchamp & Fernández‐Juricic 2005). One particular implication of living in a group is that animals become interdependent and ultimately, in the absence of group peers, the survival of an individual may be threatened. On the proximate level, prolonged social disconnection itself can be an adverse experience (Panksepp et al. 1978; Cole et al. 2015) with potentially harmful consequences (Cacioppo & Hawkley 2003; Cacioppo et al. 2011), including for example neuropsychiatric disorders (Fone & Porkess 2008; Colonnello et al. 2011; Normansell & Panksepp 2011), cardiovascular disease (Grippo et al. 2007a), neuroendocrine disruptions (Grippo et al. 2007b), suppressed immune responses (Scotti et al. 2015) and even shorter lifespans (Ruan & Wu 2008). It is no surprise therefore, that separation from group members is a strong aversive stimulus which triggers a cascade of psychological, physiological and behavioural changes (Panksepp 1998, 2005, 2011; Brunelli & Hofer 2007) priming an individual to re‐establish connections with the group (Gamallo et al. 1986; Mendoza & Mason 1986; Jones & Harvey 1987; Feltenstein et al. 2002; Apfelbeck & Raess 2008). Insofar, most studies on social separation have investigated highly social species that form relatively cohesive and stable groups (Aureli et al. 2008) throughout their lives (e.g. rats, mice). Less is known however about the implications of social separation in systems with a higher degree of fission–fusion dynamics, where individuals form temporary social groups (e.g. during nights, seasons or developmental stages), that they repeatedly leave and subsequently adhere to. Furthermore, behavioural and cognitive studies often neglect to take into account an individual's reaction to separation itself, although social disconnection clearly has a strong impact on behaviour and cognitive performance. It is therefore essential to understand what the reactions to being separated are in order to improve the accuracy of experimental data.

Common ravens (*Corvus corax*) are a highly social songbird species, characterised by a high degree of fission–fusion dynamics (Braun et al. 2012). Ravens spend their adult life in monogamous pairs that occupy territories (Rösner & Selva 2005) and defend resources therein. For the first years of life, however, ravens form temporary non‐breeder aggregations of up to several hundred individuals (Heinrich, 1989) in order to overcome the food monopolisation established by dominant territorial breeders (Marzluff & Heinrich 1991) or large predators (Stahler et al. 2002). These groups are structured on social relationships (Braun & Bugnyar 2012), with reciprocity and cooperation playing a pivotal role in dealing with the challenges of social life (Heinrich 2011). Ravens aim at establishing high‐quality relationships (Fraser & Bugnyar 2010a) that enable partners to console one another after conflicts with other conspecifics (Fraser & Bugnyar 2010b), reconcile after conflicts between them (Fraser & Bugnyar 2011) and even assist their partners in agonistic interactions (Fraser & Bugnyar 2012). Indeed, ravens pay a great deal of attention to their social environment even when they are not directly influenced by it (Massen et al. 2014). It has been shown that already in their juvenile stage, ravens’ capacity to perform in experiments is positively influenced by the presence of conspecifics (Miller et al. 2016). Separating ravens in later stages of life, as relationships develop, could thus conceivably have a greater impact on their performance. However, although social bonds are key in solving social problems, there is still a large individual variation in the number of social connections ravens have, and in how long they maintain these bonds (Braun & Bugnyar 2012). As such, separating poor socially integrated ravens (e.g. that have a low number of social allies and spend the day at a distance from other conspecifics) may induce milder reactions as compared to separating well‐integrated individuals (e.g. ravens that have a high number of social contacts or that have strong social bonds).

Stressors, whether environmental or internal, elicit in birds the secretion of glucocorticoids (primarily corticosterone) through a stepwise activation of the hypothalamic–pituitary–adrenal (HPA) axis (Siegel 1980). Measuring glucocorticoids from plasma represents a standard method for estimating the stress level of an individual (Broom & Johnson 1993), but the sample collection procedure can itself increase the amount of circulating glucocorticoids. We here use a non‐invasive and feedback‐free procedure of estimating stress, by measuring corticosterone metabolites (CM) from the faeces fraction of bird droppings (Miller et al. 1991; Palme et al. 1996; Möstl & Palme 2002). While this method is less suitable for evaluating stress levels on specific time points, due to the delay in corticosterone metabolism and its gradual accumulation in faeces, it is a useful alternative when studying longer periods of time (e.g. days), such as when determining the overall stress levels of individuals during social separation. There is substantial variability in individuals’ physiological (and behavioural) reactions to stressors, however. These differences may primarily have genetic origins (Benus et al. 1991), but can also be influenced by experiences during ontogeny (Anisman et al. 1998) and the presence of social support (de Waal & van Roosmalen 1979; Heinrichs et al. 2003; Kanitz et al. 2014). With regard to ontogenetic development, it has been shown that individuals that are hand‐reared or handled by experimenters at an early age exhibit lower levels of glucocorticoids as adults during stressful events than parent‐raised ones (Meaney et al. 1985, 1991; Hemetsberger et al. 2010). With regard to social support, there is substantial evidence showing that the presence of a partner results in a lower activation of the HPA axis after a stressful event (Cohen & Wills 1985; Rault 2012). Recently, we have also shown that social integration plays an important role in modulating stress response as ravens with more social connections are more stressed during social separation than ravens with fewer partners, and that this pattern is inversed when observing individuals under normal, group conditions (Stocker et al. 2016). Still unknown however is how ravens react behaviourally to being isolated. Handling and separating individuals for experiments most likely affects birds in different ways, and thus, it is crucial to know not only what the reactions to a stressor are, but also to determine their intensity and how long they can last.

Here, we determined the behavioural and physiological stress responses to social separation in captive non‐breeding ravens. We further investigated whether these behavioural and hormonal responses correlated. Finally, we considered the possibility of individual differences in response to social separation and we examined what life‐history characteristics might explain such variation. For this, we individually separated ravens in a compartment that was visually isolated but still in auditory range of their group of peers. During the separation period, we measured behavioural parameters from video recordings and corticosterone metabolites (CM) from droppings. We expected that in the beginning of the separation, all individuals would show a peak in CM and distress behaviours due to the handling procedure. We predicted that at the end of the separation period, we should find pronounced individual differences in behavioural and hormonal (CM) patterns, as they likely reflect the ravens’ capacity to cope with being socially isolated. In general, we predicted a positive correlation between the intensity of behavioural responses and CM levels. However, we speculated that individuals would show varying combinations of vocal, self‐directed and environment‐directed reactions, owing to the individual's raising method and social integration. Specifically, we expected that hand‐raised individuals and/or poorly integrated ones would exhibit lower CM levels and behaviours indicative of a calm state, and that parent‐raised and/or well‐integrated individuals would have more difficulties in coping with the separation, with higher levels of CM and more behaviours indicating agitation.

## Methods

### Animals and Housing

We tested a total of 25 subadult ravens (14 males, 11 females) that originated from several different breeding pairs from research stations, zoos and private owners throughout Europe. A total of 15 ravens (10 males, five females) were collected as nestlings and hand‐raised to fledging by humans; 10 ravens (four males, six females) were parent‐raised and collected 4 mo post‐fledging. Ravens were kept in three social groups comprised of eight birds (Group A), seven birds (Group B) and 10 birds (Group C, see Table 1), during three consecutive years. The group structure simulated natural, non‐breeder social groupings (Marzluff et al. 1996; Stahler et al. 2002). Ravens were individually marked with coloured leg bands for identification and were kept on a food diet composed of meat, eggs, vegetables, dairy products, bread and phytobiotics. Water was available *ad libitum*.

**Table 1:** Overview of test subjects in testing order

Group	Test period	ID	Sex	Year of hatch	Origin	Raising method
A	Dec 2011–May 2012	Heidi	F	2010	Innsbruck, Austria	Parent‐raised
		Lena	F	2010	Klosterneuburg, Austria	Parent‐raised
		Anton	M	2010	Innsbruck, Austria	Parent‐raised
		Elen	F	2010	Bayerischer Wald, Germany	Parent‐raised
		Jonas	M	2010	Wels, Austria	Parent‐raised
		Klara	F	2010	Bayerischer Wald, Germany	Parent‐raised
		Jakob	M	2010	Bayerischer Wald, Germany	Parent‐raised
		Sophie	F	2010	Klosterneuburg, Austria	Parent‐raised
B	Dec 2012–May 2013	Thor	M	2011	Grünau im Almtal, Austria	Parent‐raised
		Orm	M	2011	Lund, Sweden	Hand‐raised
		Lellan	F	2011	Lund, Sweden	Hand‐raised
		Ray	M	2011	Lund, Sweden	Hand‐raised
		Matte	M	2011	Lund, Sweden	Hand‐raised
		Astrid	F	2010	Wels, Austria	Hand‐raised
		Skadi	F	2011	Grünau im Almtal, Austria	Parent‐raised
C	Dec 2013–May 2014	George	M	2012	Stockholm, Sweden	Hand‐raised
		Laggie	M	2012	Bayerischer Wald, Germany	Hand‐raised
		Louise	F	2012	Stockholm, Sweden	Hand‐raised
		Rufus	M	2012	Korneuburg, Austria	Hand‐raised
		Adele	F	2012	Bayerischer Wald, Germany	Hand‐raised
		Horst	M	2012	Stockholm, Sweden	Hand‐raised
		Nobel	F	2012	Stockholm, Sweden	Hand‐raised
		Tom	M	2012	Bayerischer Wald, Germany	Hand‐raised
		Paul	M	2012	Wels, Austria	Hand‐raised
		Max	M	2012	Wels, Austria	Hand‐raised

**Figure 1:**
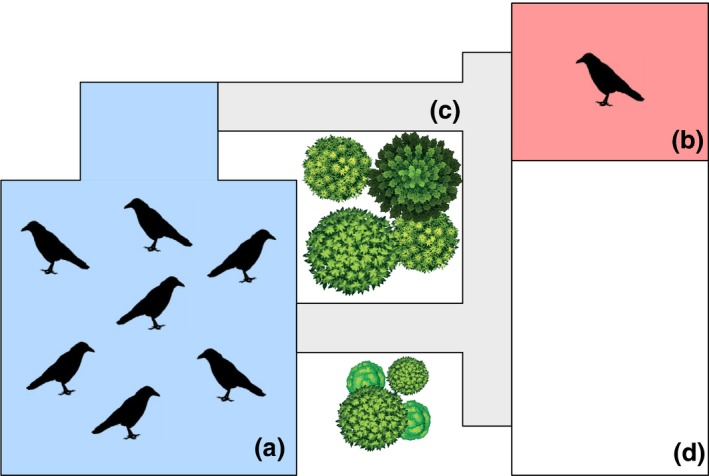
Experimental corvid aviary complex at Haidlhof Research Station, Austria. (a) main aviary (15 × 15 × 3.75 m), (b) test compartment (10 × 8 × 4.25 m), (c) connecting runways, (d) unused adjacent compartment (16 × 10 × 4.25 m). [Colour figure can be viewed at wileyonlinelibrary.com]

We conducted our study at the Haidlhof Research Station, Bad Vöslau, Austria – a joint research site of the University of Vienna and the University of Veterinary Medicine Vienna. The aviary complex for corvids consists of three compounds. For this study, we used the middle compound for keeping (main aviary, with a usable volume of approximately 850 m^3^, Fig. 1a) and one compartment (340 m^3^) of the right compound for separation (Fig. 1b). Both aviaries were partitioned and equipped with natural structures (e.g. wood, rocks, gravel, sand) and artificial objects (e.g. food bowls, bathing pools, toys) so as to ensure welfare, optimal behaviour expression (e.g. bathing, food and object caching, conflict escape possibilities) and to provide protection during extreme weather conditions. The main aviary and the separation compartment were visually isolated, but within acoustic range.

## Experimental Procedure

We carried out the experiment throughout the winter–spring season of three consecutive years (2012, 2013, 2014, see Table 1). We kept a seasonal consistency taking into account age of subjects (i.e. 1½ years at the start of the season; see Table 1) and possible interseasonal variability in CM excretion pattern (Kotrschal et al. 1998, 2000; Stöwe et al. 2008). The winter–spring season coincides with the pre‐ and breeding seasons for adult ravens; for subadult, non‐breeding ravens, this is a period of high social activity, when a dominance rank hierarchy is being established (Loretto et al. 2012) and birds are focused on consolidating their position in the hierarchy (e.g. by displaying dominance towards subordinates), strengthening social bonds (e.g. by selective allopreening, spending time in close proximity to and sharing food with preferred individuals) and attempting to form alliances (e.g. by providing selective support in agonistic interactions). We thus expected that separating individuals during this highly social season would have a significant impact on behavioural and hormonal expression.

**Figure 2:**
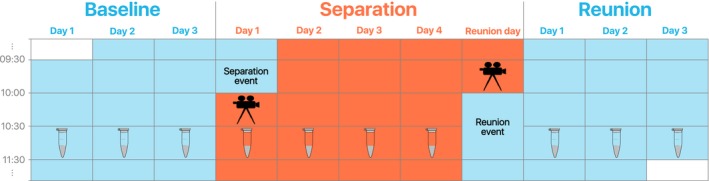
Procedure outline. The experiment consisted of one test stage (‘separation’) and two control stages (‘baseline’ and ‘reunion’). Droppings were collected every day, except on the reunion day. Video recordings were taken after the start and before the end of the separation period. [Colour figure can be viewed at wileyonlinelibrary.com]

We initiated the separation event by calling the focal subject into a small compartment of the main aviary (Fig. 1a) where an experienced person, unfamiliar to the ravens, quickly caught it with a net. We then transferred the bird to the separation compartment (Fig. 1b) where it was allowed to habituate to the novel enclosure for 15 min. After this, we video‐recorded the subject for a period of 10 min, at the same time keeping note of excreted droppings. We then proceeded inside the separation compartment to collect samples of its droppings (for a total of 1.5 h following the separation event). We collected droppings from the subject on each day of the three experiment stages (i.e. baseline, separation and reunion, Fig. 2), with the exception of the reunion day. We did not collect droppings on this day because the reunion event overlapped with the regular collection time, and we did not want to influence the behaviour of birds by being physically present inside the aviary. Each day we collected multiple samples per individual ($\overset{¯}{x}$ ± SD = 2.94 ± 1.09) to control for interindividual variations in excreted corticosterone metabolites (Touma & Palme 2005). At the end of the separation stage (i.e. before the reunion event, Fig. 2), we video‐recorded the subject for another 10 min. Between consecutive individual separations, we kept a 14‐d break interval to allow ravens to re‐establish social connections that were potentially disturbed by our experimental manipulation. For the first 2 yrs, we separated birds for a period of 4 d, with 3 d before and after the separation stage. In the third year, due to logistical constraints, we reduced each stage of the experiment (i.e. before, during and after the separation) by 2 d, but kept all other aspects of the experimental procedure identical.

### Study Approval, Ethics Statement and Note

Our study was approved by the Austrian Federal Ministry of Science, Research and Economy (approval number: BMWFW‐66.006/0016‐WF/II/3b/2014) and by the ethical board of the behavioural research group of the Faculty of Life Sciences, University of Vienna (Nr: 2015–003a). After the study ended, subjects remained in captivity at the Haidlhof Research Station for further research.

Note that this study has been part of a large project on individual separation and subgroup manipulation of non‐breeding ravens at Haidlhof Research Station. As such, a part of the data set presented here (i.e. CM values for the first and second periods of testing) has been used in a recent publication (Stocker et al. 2016). The present study draws on a much larger data set, (1) reporting for the first time the behaviour during separation and (2) including an additional third testing period (with new data from 10 hand‐raised birds).

### Hormonal Analyses

Droppings were frozen at −20°C until analysis. For the CM extraction, 0.1 g of wet dropping and 1 ml 60% methanol were mixed, shaken for 30 min and centrifuged for 15 min at 1942 ***g*** (Palme et al. 2013). If droppings weighed <0.1 g, the volume of methanol was rescaled accordingly. The resulting extract was diluted with assay buffer (1:5) and analysed with an 11‐oxoetiocholanolone assay (using 11‐oxoetiocholanolone as standard and antibodies raised in rabbits against 5ß‐androstane‐3α‐ol‐11,17‐dione‐17‐CMO: bovine serum albumin; sensitivity: 1 ng CM/g dropping; for a detailed assay description see Möstl et al. 2002). This assay has been previously validated biologically (Stöwe et al. 2008) and physiologically (Stocker et al. 2016). All samples were analysed in duplicates. Values outside the range (i.e. >2*SD) were subsequently excluded. The interassay coefficient of variance (CV) of the separations in 2012 and 2013 were 10.1% and 7.5%, respectively, and the intra‐assay CV was 5.7%.

### Video Analysis

We video‐recorded the first 10 min after the habituation period (i.e. a 15‐min period following the separation event) and the last 10 min before the reunion event. We chose these two phases as being representative time frames of the individual's behavioural repertoire, first when being submitted to an acute stressor – that of being physically manipulated – and second, when several days had passed with no contact to its group peers. An extended observation period would not have necessarily resulted in more precise behavioural measures and could have in fact diluted the response to being handled for separation. The time gap between the two phases increases the likelihood that the behaviours of the second phase would in fact be indicative of a chronic stress state due to being separated from, although in close proximity to, the group. We coded behavioural parameters using Solomon Coder v. 12.02 (András Péter, www.solomoncoder.com), and measured the frequency and duration of the following behavioural categories: locomotion, vocalisations, self‐, object‐ and structure‐directed behaviours (see Table S1). In a succession of same repeated behaviours, we considered each parameter as one behavioural event if a break of more than 5 s elapsed in between. An exception to this rule is represented by ‘calls’ that we defined as bouts of three to five utterances with typical breaks of 2–3 s. We defined ‘song’ as a collection of vocalisations (including calls) with no clear temporal or acoustical demarcation. We defined ‘immobility’ as the lack of movement and any other behaviour, except scanning motions of the head. We additionally looked at the time spent in three different aviary height levels: high, middle and low, defined by the perching structures available at that particular height division (high: >3 m; middle: 1.5–3 m; low: <1.5 m above ground).

### Statistical Data Analysis

We used a principal component analysis (PCA) to reduce the amount of behavioural response variables (Tabachnick & Fidell 2007). We considered coefficients of correlation >0.5 or smaller than −0.5 to be high loadings. We used a varimax rotation with Kaiser normalisation to minimise the number of variables that have high loadings on each component, and to simplify the interpretation of the components. Based on comparisons of Kaiser–Meyer–Olkin measures of adequacy on both the overall and individual behavioural level, we included a total of eight behavioural variables in the PCA (see Table 2) that reflected an individual's behavioural repertoire during social separation. We analysed the two sets of behaviours (at the beginning and the end of the separation stage, Fig. 2) together so as to prevent an *a priori* presumption of difference and to avoid any subsequent PCA bias.

**Table 2:** Variables included in the principal component analysis

Variable name	Variable description
Immobile	Duration spent immobile and inactive (i.e. producing no behaviour except scanning movements of the head)
Walking and hopping	Duration spent walking and/or hopping on structures and ground
Vocalising	Duration spent calling and singing
Feeding and drinking	Duration spent feeding or drinking water
Manipulating object	Duration of object manipulation
Manipulating structure	Frequency of structure pecking
Rousing	Frequency of rousing (i.e. shaking body) behaviours
Ground level	Duration spent on ground or ground structures

We ran generalised linear mixed models (GLMMs) on the extracted regression scores for each of the three principal components (PCs). As fixed factors, we included the phase of separation (i.e. first and last day within the separation stage), and additionally looked at the influences of the subject's sex, its raising method (n = 15 hand‐ vs. n = 10 parent‐raised), its social integration (i.e. subject integration relative to the individual with highest social integration value within the group), the length of time spent in separation (i.e. if the period between the separation event and the reunion event was either two, n = 10, or 4 d, n = 15) and the interactions of these factors with phase. We entered subject identity as a random factor. From our PCs, we excluded two outliers that exceeded 4*SD (one individual in component 1, and another individual in component 3, see supporting information). Thus, from the initial sample size of 50, the models on PC1 and PC3 were reduced to a sample size of 48, while the analysis on PC2 was run on the full data set. We used GLMMs with a normal distribution and an identity link function.

We checked for differences in CM changes over the baseline stage between the first and last days of separation, using a nonparametric Wilcoxon signed‐rank test.

Further, we checked for possible links between behavioural and hormonal changes. We first checked whether hormonal changes could predict behaviour. For this, we split the initial PCs between the first and last separation days and ran two separate sets of GLMMs on the resulting PCs (i.e. continuous response variable). We calculated the CM changes over baseline values (i.e. mean CM concentration of the day(s) prior to the separation event) of the first and last full days of separation (see Fig. 2) and used this ΔCM data in our models, as a fixed factor. We included as additional fixed factors: subject's sex, raising method and the interactions of ΔCM with sex and raising method. We entered subject identity as a random factor. We then checked whether behaviour could predict hormonal changes. For this, we ran GLMMs on the relative (to baseline) CM concentration of the second full day of separation (i.e. continuous response variable). As fixed factors, we included subject's sex, its raising method (hand‐ vs. parent‐ raised), PC scores and PC interactions with sex and raising method. Again, we entered subject identity as a random factor.

We calculated all GLMMs using a backward stepwise elimination based on AICc. Starting with the full model that included all fixed factors and (relevant) interactions, we dropped factors from the model step by step if their removal lead to a lower AIC value, and thus improved the model fit. For clarity, we here report only the results of the best‐fitting models. For the actual best‐fitting models, see the supporting information (Tables S2–S4). We controlled for the goodness of the GLMM fit by ensuring that the residuals were normally distributed and did not vary significantly between individuals.

We determined the social integration of an individual by looking at the number of interaction partners it has and how frequently they were found in close proximity (i.e. one body length) of each other. We extracted data from regular observations throughout the testing periods, but not during the experiment days. With this data, we constructed a social network that returned absolute values for the degree of interactions. To have comparable values between groups, we then determined the relative social integration value against the individual with the highest social integration score in each group. Note that we only had data on social integration for the first two groups tested and that this data and more detailed explanations on the method are published in Stocker et al. 2016. In our present study, when we ran models with social integration included, model reductions revealed that social integration was not present in the best‐fitting models. Furthermore, social integration reduced our subject sample size (n = 15). As such, in further analyses, we decided to exclude social integration as a fixed factor in our models, again increasing our subject sample size to the original number (n = 25).

All analyses were conducted in SPSS v21, all tests were two‐tailed and alpha was set at 95%.

## Results

### Behavioural and Hormonal Responses to Social Separation

We narrowed down the behavioural variables using a PCA. With an eigenvalue of minimum 1.0 and scree plot investigations, we extracted three PCs, explaining a cumulative behavioural variance of 80.25% (see Table 3). Based on the variable loadings, we found PC1 to be associated with behaviours reflective of a calm state, hence hereafter referred to as ‘comfort’. The two behaviours loading on PC2, namely vocalising and being immobile, loaded inversely to one another, and therefore raised the possibility of subjects presenting different behavioural patterns of coping with the separation. We therefore refer to this component as ‘coping pattern’. The behaviours loading on PC3 were indicative of agitated behaviours and therefore we named it ‘tension’.

**Table 3:** Rotated principal component matrix showing loadings (>0.5) for behavioural variables on the extracted PCs

Variable	Component			Communality h^2^
	1 ‘comfort’	2 ‘coping pattern’	3 ‘tension’	
Immobile		0.908		0.887
Walking and hopping	0.887			0.896
Vocalising		−0.935		0.920
Eating and drinking	0.821			0.733
Manipulating object	0.739			0.554
Manipulating structure			0.819	0.777
Rousing			0.750	0.778
Ground level	0.912			0.875
Eigenvalue	2.974	2.018	1.429	
% variance explained	37.171	25.225	17.863	

**Figure 3:**
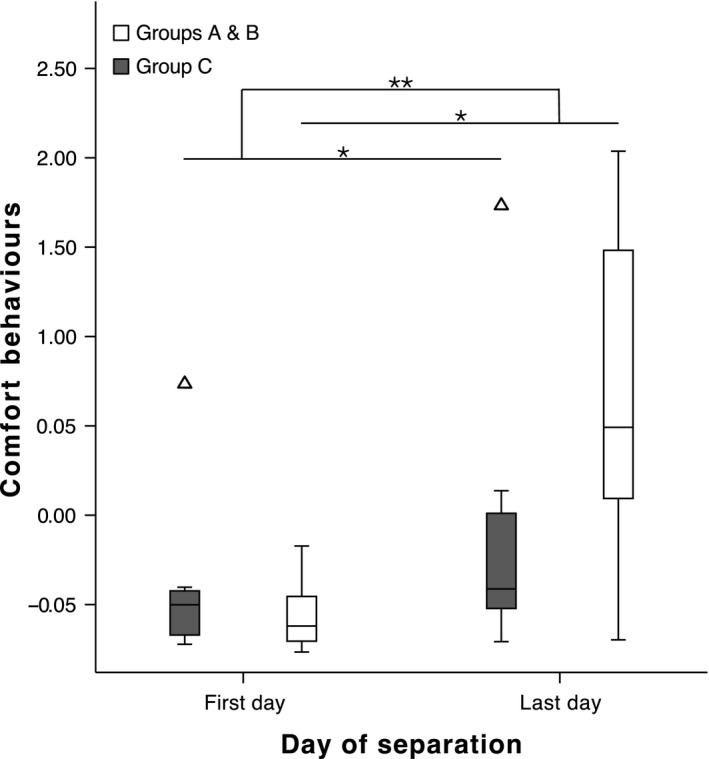
‘Comfort’ on the first and last days of separation. Median ± Quartiles and 95% confidence intervals of ‘comfort’ behaviours for groups A and B (4‐d separation, n = 15, white bars) and group C (2‐d separation, n = 10, grey bars). GLMM: *p < 0.05; **p < 0.01.

**Figure 4:**
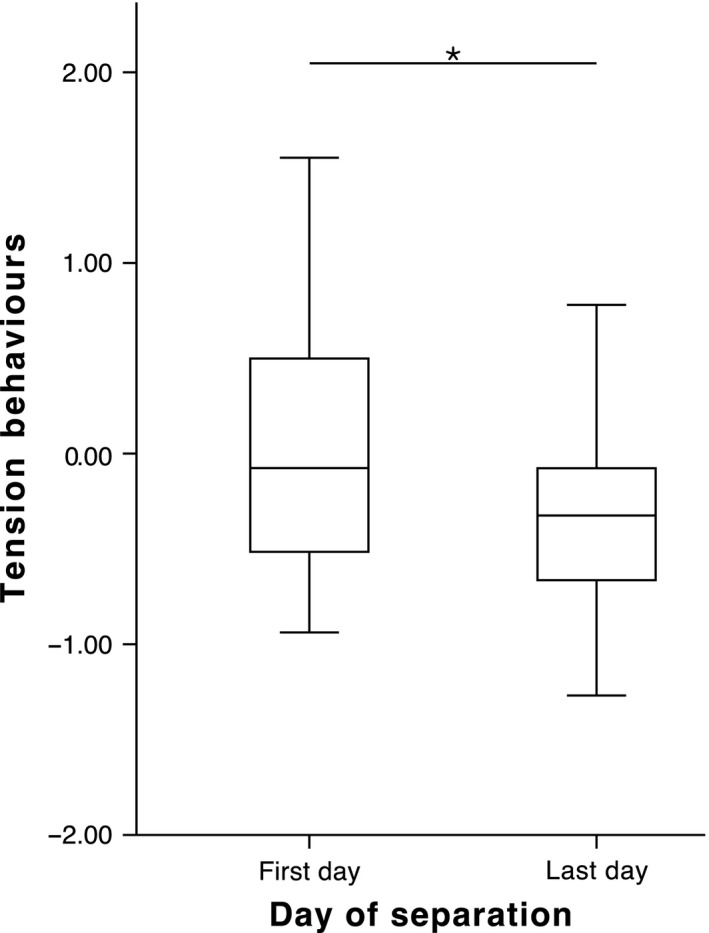
‘Tension’ on the first and last days of separation. Median ± Quartiles and 95% confidence intervals of ‘tension’ behaviours for all groups (n = 25). GLMM: *p < 0.05.

As predicted, the behavioural components were affected by the phase of separation. ‘Comfort’ increased, that is there was a positive difference between the last and first days of the separation stage (GLMM: β = −1.049, *F*_1,42_ = 9.804, p = 0.003; Fig. 3), and we found a significant interaction between phase with the length of separation time (GLMM: β = −1.048, *F*_2,42_ = 4.279, p = 0.02; Fig. 3); that is, when animals were kept in a longer separation, the scores were significantly higher for this component (Wilcoxon signed‐rank test: *Z* = −3.351, p = 0.001). ‘Tension’ decreased, that is there was a negative difference between the last and first days of the separation (GLMM: β = 0.926, *F*_1,42_ = 4.214, p = 0.046; Fig. 4). We found no interaction effect between any of the fixed parameters for ‘tension’.

**Figure 5:**
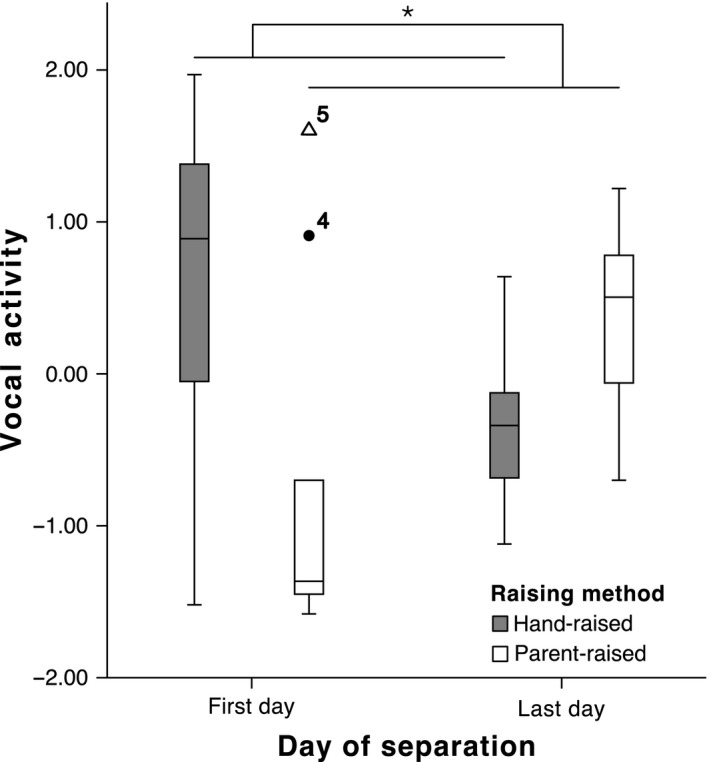
Vocal activity on the first and last days of separation. Median ± Quartiles and 95% confidence intervals of vocalisations for hand‐raised ravens (n = 15, grey bars) and parent‐raised ravens (n = 10, white bars). GLMM: *p < 0.05.

Unexpectedly, PC2 ‘coping pattern’ showed an interaction effect of phase with raising method (GLMM: β = −2.073, *F*_1,42_ = 8.924, p = 0.005, Fig. 5): Vocalisations decreased in favour of immobility for hand‐raised ravens (n = 15, Wilcoxon signed‐rank test: *Z* = −2.442, p = 0.015), while the opposite shift tended to occur for parent‐raised ravens (n = 10, Wilcoxon signed‐rank test: *Z* = −1.886, p = 0.059).

Contrary to our expectation, we found no differences in between the first and last full days of separation regarding ΔCM over baseline (Wilcoxon signed‐rank test: *Z* = −1.164, p = 0.244). A summary of CM concentration means per individual, group and experimental stage is presented in Table 4.

**Table 4:** Average CM concentrations per experimental stage

Group	Subject	Average CM concentration (ng/g)				
		Baseline	Separation			Reunion
			First day	Last day	Average separation	
A	Heidi		178.01	119.79	212.91	62.73
A	Lena	123.35	205.65	188.21	284.09	97.64
A	Anton	112.94	518.05	90.60	202.85	101.33
A	Elen	53.19	113.97	110.99	125.88	105.44
A	Jonas	162.67	142.74	134.38	109.14	277.75
A	Klara	68.74	146.14		92.92	75.85
A	Jakob	44.62	150.81	113.65	146.16	115.24
A	Sophie			215.75	182.91	161.59
Group A average		94.25	207.91	139.05	169.61	124.69
B	Thor	497.83		418.36	444.10	286.28
B	Orm	789.12	901.58	823.90	508.74	457.53
B	Lellan	151.91	702.82		363.10	507.36
B	Ray	131.58	339.30	158.21	228.58	206.55
B	Matte	377.52	250.03	150.68	192.72	319.70
B	Astrid	715.36	261.25	316.71	437.93	641.45
B	Skadi	540.85	390.67	245.12	264.32	291.26
Group B average		457.74	474.28	352.16	348.50	387.16
C	George	54.56	51.21	167.42	358.07	159.61
C	Laggie	84.15	58.56	273.50	225.47	48.90
C	Louise	94.00	17.65	48.36	29.94	42.55
C	Rufus	324.56	38.23	48.84	96.73	53.13
C	Adele	24.41	20.82	136.46	62.48	29.04
C	Horst		50.94	194.73	107.63	141.90
C	Nobel	41.97	36.25	33.48	33.08	70.57
C	Tom		59.58	572.85	222.34	20.81
C	Paul	80.74	110.00	126.83	103.21	161.15
C	Max	175.04	359.33	107.91	203.22	
Group C average		109.93	80.26	171.04	144.22	80.85
All groups average		221.39	221.89	208.55	209.54	184.81

### Links Between Behavioural and Hormonal Responses

We tested whether changes in behaviour (PC 1‐3) correlated with hormonal changes, but did not find any significant correlations. The relative (to baseline) CM values of the first day of separation could not be explained by the three PCs of that same day: the best‐fitting model on ‘comfort’ only included the intercept; the best‐fitting model on ‘coping pattern’ did not include CM values, but reiterated a raising effect we found in previous analyses (GLMM: β = −1.461, *F*_1,22_ = 9.702, p = 0.005); the best‐fitting model on ‘tension’ did not show any significant effect. Similarly, relative (to baseline) CM values of the last full day of separation did not predict the behaviours (PC 1‐3) of the last day of separation (i.e. before reunion with the group): the best‐fitting model on ‘comfort’ did not show any significant effect; the best‐fitting model on ‘coping pattern’ did not include CM values, but reiterated a raising effect we found in previous analyses (GLMM: β = 0.676, *F*_1,23_ = 9.276, p = 0.006); the best‐fitting model on ‘tension’ only included the intercept. Finally, behaviours (PC 1‐3) on the separation day did not predict the relative (to baseline) CM values of the second day of separation (see Tables S5–S11).

Based on the differences we found in the behavioural data between hand‐raised and parent‐raised ravens, we ran *post hoc* analyses to test for differences in CM concentrations, with regard to the raising method. We found a non‐significant trend for parent‐raised ravens to show a higher CM increase over baseline [mean baseline concentration of the day(s) prior to the separation event] on the separation day (*X *± SE = 121.47 ± 35.45 ng) than hand‐raised ravens (*X *± SE = 26.51 ± 38.81 ng, Mann–Whitney *U*‐test: *U *=* *23, *N*1 = 7, *N*2 = 13, p = 0.081).

## Discussion

With the present research, we aimed at determining how individual ravens deal with an artificially induced separation from their social group, both in terms of behavioural responses and hormonal patterns. We expected the separation event to be an intense stressor that would produce a distressed state from which we could identify and extract the behaviours linked with it. Indeed, we found that, after being taken out of the group, ravens pecked at structures (e.g. branches and walls) and frequently roused – agitated behaviours that decreased on the last separation day (Fig. 4). Meanwhile, comfort behaviours were frequently produced at the end compared to the beginning of the separation (Fig. 3). Ravens demonstrated a calm state by spending more time on the ground, more time feeding and drinking and more frequently manipulating objects. Further supporting the progressive instalment of a state of relaxation was given by the influence of separation period length (Fig. 3), whereby individuals that underwent a 2‐d separation did show an increase in relaxation behaviours, but on a smaller scale than ravens that went through a 4‐d separation.

In ravens, vocal activity and mobility seem to correlate, as ravens called and singed when they were not immobile (Table 3, Fig. 5). The fact that there was a contrasting difference between hand‐raised ravens (i.e. that produced a high amount of vocalisations in the beginning and became immobile in the end) and parent‐raised ravens (i.e. initially immobile and finally vocally active) could be explained by an influence of early‐life experiences. The ontogenetic background is in fact one aspect that influences individual variability (Levine 1994; Meerlo et al. 1999; Koolhaas 2008), and many laboratory experiments using mice and rats include strains of animals with distinct reactivity to stress, strains that were selected by raising individuals under different environmental conditions (Koolhaas et al. 2007). Hand‐raising itself exposes animals to human presence and activity early on in life, and as such, any interaction with humans is more easily tolerated (Leussis & Bolivar 2006). Because our hand‐raised ravens were trained from an early age to participate in short‐term group or individual experiments and to cooperate with human experimenters, it may be that the event of being caught, handled and translocated into an isolated compartment was more tolerable for them and did not represent a strong initial stressor. However, they were not used to being separated from their peers for a longer period of time (e.g. overnight) and this prolonged isolation could have been a more stressful situation, explaining the behavioural progression to immobility on the last day of the separation stage. These two behavioural axes are thus likely an expression of different arousal states (i.e. immobile when highly aroused and vocalising when low aroused). This interpretation is supported, to a limited extent, by the difference in CM concentration increase between hand‐ and parent‐raised individuals on the first day of separation, even though just as a trend. In a study on chicks separated from their peer group, authors similarly found a moderate positive correlation between the number of distress vocalisations produced and CM levels (Feltenstein et al. 2003).

With our experiment, we were able to elicit separation distress behaviour in ravens, but, contrary to our predictions, we were unable to connect these behaviours with physiological responses. This result could have several causes. Firstly, our experimental setup may have been imperfect. We separated each raven in a compartment where it remained visually isolated from the rest of its group peers, but could still hear and call to them. Remaining in auditory range during separation perhaps decreased the intensity of hormonal stress responses. Secondly, the timing of collected droppings and recorded behavioural data may not have accurately reflected one another. According to the physiological validation, in ravens, the highest CM peak would occur between one and one and a half hours after a stressful event (Stocker et al. 2016). Even so, the HPA‐axis activation due to the separation event may have differed among individuals, occurring sooner in some and more delayed in others. We thus might have missed the full response in some of the subjects. Furthermore, social integration may play a key role in disentangling hormonal responses. We were previously able to show a significant effect of social integration on the HPA‐axis reactivity of separated individuals between the three stages of separation: in comparison with the group situation, well‐integrated individuals were more stressed during separation, while poorly integrated individuals seemed to be more relaxed during separation (Stocker et al. 2016). However, when looking just at the separation stage, social integration was not present in our best‐fitting models, and by excluding it from subsequent analyses, we were unable to find any difference in hormonal patterns over the separation period. What we did find unexpectedly was an effect of rearing method on behavioural activity. Perhaps the intertwining of social integration and raising method primed some birds to react more to handling stress and others more to social isolation stress, averaging out the hormonal response to separation. Given our experimental design, we cannot fully exclude the possibility that non‐social, less complex factors (e.g. the physical environment) influenced the birds’ responses to the experimental manipulations. Future research is required to disentangle the effects of rearing method and social integration on behavioural and physiological responses to stress in ravens, paying attention to a better match between hormonal and behavioural data sampling and having an experimental design that excludes potential reactions to the immediate environment.

Taken together, in our study, we found that ravens show clear behavioural responses to being individually separated from the group. These behaviours were most likely determined by different arousal states and were mainly affected by the ravens’ early‐life experience, specifically hand‐raising. Unfortunately, what controls these arousal states and how the birds cope with them remains an open issue. Based on our findings, we propose that different processes: social integration, rearing method, distance to group peers and length of separation may work simultaneously in regulating arousal states and thus in generating different patterns of behavioural and physiological reactions to social stress. These results are relevant to the high plasticity shown in individual settings, which appears to contrast from individually consistent patterns in raven social groups (Miller et al. 2016). These findings merit further investigation, in particular to see how the many facets of sociality shape individual strategies in group‐living non‐human animals, paying special attention to the possible influence of early‐life experiences.

## Supporting information


**Table S1.** Behavioural parameters.
**Table S2.** Best fitting model on PC1.
**Table S3.** Best fitting model on PC2.
**Table S4.** Best fitting model on PC3.
**Table S5.** Best fitting model (=null model) on PC1 on the first day of separation.
**Table S6.** Best fitting model on PC2 on the first day of separation.
**Table S7.** Best fitting model on PC3 on the first day of separation.
**Table S8.** Best fitting model on PC1 on the last day of separation.
**Table S9.** Best fitting model on PC2 on the last day of separation.
**Table S10.** Best fitting model on PC3 of the last day of separation.
**Table S11.** Best fitting model on hormonal values of the second day of separation.
