# Supplementary material for: Behavioural and Hormonal Stress Responses to Social Separation in Ravens, Corvus corax
Source: Ethology. 2016 Dec 28;123(2):123–35. doi: 10.1111/eth.12580 (PMC5299477; doi:10.1111/eth.12580)
Supplement: Supplementary file 1 — Table S1. Behavioural parameters. Table S2. Best fitting model on PC1. Table S3. Best fitting model on PC2. Table S4. Best fitting model on PC3. Table S5. Best fitting model (=null model) on PC1 on the first day of separation. Table S6. Best fitting model on PC2 on the first day of separation. Table S7. Best fitting model on PC3 on the first day of separation. Table S8. Best fitting model on PC1 on the last day of separation. Table S9. Best fitting model on PC2 on the last day of separation. Table S10. Best fitting model on PC3 of the last day of separation. Table S11. Best fitting model on hormonal values of the second day of separation. [file ETH-123-123-s001.docx]

**Supplementary material to:**

**Behavioural and hormonal stress responses to social separation in ravens, *Corvus corax***

**Alexandru M. Munteanu, Martina Stocker, Mareike Stöwe,**

**Jorg J. M. Massen, Thomas Bugnyar**

# Material and Methods

**Table S1.** Behavioural parameters

| **Category** | **Parameter** | **Definition** |
| --- | --- | --- |
| Locomotion | flying | moving through the air making use of wings |
|  | walking and hopping | moving by lifting and setting each foot in turn or both feet off the ground, but not flying |
|  | immobile | not moving from one position (and not producing any other behaviour, except scanning movements of the head) |
| Vocalisations | call | short, often repeated sounds |
|  | song | long vocalisations, usually containing a collection of calls |
| Self-directed | auto-preening | self touching feathers with the beak |
|  | scratching | self scratching by using the foot |
|  | rousing | momentarily shaking/rousing feathers and subsequent smoothing them down |
|  | stretching | slowly extending wing(s) and/or one leg |
|  | beak wiping | rubbing beak on structure (e.g. branch) |
| Manipulations | feeding & drinking | the act of eating or drinking |
|  | manipulating object | using beak to manipulate a small or portable object |
|  | pecking structure | pecking with beak on big, non-portable objects |

# Results

**Behavioural and hormonal responses to social separation**

Regarding PC1, original analyses revealed that the full model was the best-fitting model and found an effect of phase of separation (GLMM: β = -1.347, *F_1,42_*=9.034, p=0.004). However, we noticed an outlier that, after checking the raw data, exceeded 4*SD. Therefore, we re-ran our analyses without this subject.

Regarding PC3, original analyses indicated also that the full model was the best-fitting model, but did not show any significant effect. We excluded an outlier (exceeding 4*SD, different than the one in PC1) and we ran the analyses again.

## Links between behavioural and hormonal responses

Regarding PC1 original analyses and analyses where we excluded the PC1 outlier yielded the same results.

Regarding PC3, original analyses indicated a raising effect (GLMM: β = -0.791, *F_1,17_* = 4.586, p=0.047), but did not show any significant effect of hormonal increase over baseline. After excluding the PC3 outlier and running the analyses again, the raising effect disappeared.

## Best fitting models on PCs

**Table S2.** Best fitting model on PC1

| **Variable** | **β** | **F _df1, df2_** | **p** |
| --- | --- | --- | --- |
| Intercept | 0.504 | 6.040 _5, 42_ | **0.000** |
| Phase of separation | - 1.049 | 9.804 _1, 42_ | **0.003** |
| Length of separation * Phase of separation | - 1.048 | 4.279 _2, 42_ | **0.020** |
| Raising method * Phase of separation | - | 0.660 _2, 42_ | 0.522 |

**Table S3.** Best fitting model on PC2

| **Variable** | **β** | **F _df1, df2_** | **p** |
| --- | --- | --- | --- |
| Intercept | - 0.225 | 2.927 _7, 42_ | **0.014** |
| Length of separation | - 0.305 | 0.836 _1, 42_ | 0.366 |
| Raising method | 0.823 | 0.378 _1, 42_ | 0.542 |
| Sex | - 0.210 | 0.453 _1, 42_ | 0.504 |
| Phase of separation | 1.114 | 0.131 _1, 42_ | 0.719 |
| Length of separation * Phase of separation | - 0.018 | 0.001 _1, 42_ | 0.979 |
| Raising method * Phase of separation | - 2.073 | 8.924 _1, 42_ | **0.005** |
| Sex * Phase of separation | 0.067 | 0.017 _1, 42_ | 0.898 |

**Table S4.** Best fitting model on PC3

| **Variable** | **β** | **F _df1, df2_** | **p** |
| --- | --- | --- | --- |
| Intercept | - 0.422 | 1.685 _5, 42_ | 0.159 |
| Phase of separation | 0.926 | 4.214 _1, 42_ | **0.046** |
| Raising method * Phase of separation | - | 1.550 _2, 42_ | 0.224 |
| Sex * Phase of separation | - | 1.325 _2, 42_ | 0.277 |

## Best fitting models for hormonal correlates with behaviour

**Table S5.** Best fitting model (= null model) on PC1 on the first day of separation

|  | **β** | **t** | **p** |
| --- | --- | --- | --- |
| Intercept | -0.505 | -2.229 | **0.036** |

**Table S6.** Best fitting model on PC2 on the first day of separation

| **Variable** | **β** | **F _df1, df2_** | **p** |
| --- | --- | --- | --- |
| Intercept | 0.087 | 5.014 _2, 22_ | **0.016** |
| Raising method | -1.461 | 9.702 _1, 22_ | **0.005** |
| Sex | - 0.124 | 0.072 _1, 22_ | 0.791 |

**Table S7.** Best fitting model on PC3 on the first day of separation

| **Variable** | **β** | **F _df1, df2_** | **p** |
| --- | --- | --- | --- |
| Intercept | 0.474 | 1.246 _2, 16_ | 0.314 |
| Raising method | -0.560 | 2.079 _1, 16_ | 0.169 |
| CM First Day increase over baseline | -0.002 | 1.427 _1, 16_ | 0.250 |

**Table S8.** Best fitting model on PC1 on the last day of separation

| **Variable** | **β** | **F _df1, df2_** | **p** |
| --- | --- | --- | --- |
| Intercept | 0.806 | 1.317 _3, 15_ | 0.306 |
| Sex | -0.436 | 1.162 _1, 15_ | 0.298 |
| Raising method | -0.443 | 1.237 _1, 15_ | 0.284 |
| CM Last Day increase over baseline | -0.001 | 1.803 _1, 15_ | 0.199 |

**Table S9.** Best fitting model on PC2 on the last day of separation

| **Variable** | **β** | **F _df1, df2_** | **p** |
| --- | --- | --- | --- |
| Intercept | -0.351 | 9.276 _1, 23_ | **0.006** |
| Raising method | 0.676 | 9.276 _1, 23_ | **0.006** |

**Table S10.** Best fitting model on PC3 of the last day of separation

| **Variable** | **β** | **t** | **p** |
| --- | --- | --- | --- |
| Intercept | -0.314 | -0.785 | 0.440 |

**Table S11.** Best fitting model on hormonal values of the second day of separation

| **Variable** | **β** | **F _df1, df2_** | **p** |
| --- | --- | --- | --- |
| Intercept | 381.291 | 0.450 _11, 9_ | 0.894 |
| Sex | 949.733 | 0.437 _1, 9_ | 0.525 |
| Raising | -1743.368 | 0.978 _1, 9_ | 0.348 |
| PC1 1^st^ day | 344.887 | 0.067 _1, 9_ | 0.802 |
| PC2 1^st^ day | -34.061 | 1.558 _1, 9_ | 0.243 |
| PC3 1^st^ day | -118.828 | 0.726 _1, 9_ | 0.416 |
| Sex * PC1 1^st^ day | 1850.906 | 0.569 _1, 9_ | 0.470 |
| Sex * PC2 1^st^ day | 109.664 | 0.361 _1, 9_ | 0.563 |
| Sex * PC3 1^st^ day | -390.012 | 0.498 _1, 9_ | 0.498 |
| Raising * PC1 1^st^ day | -2982.451 | 1.027 _1, 9_ | 0.337 |
| Raising * PC2 1^st^ day | -327.679 | 2.297 _1, 9_ | 0.164 |
| Raising * PC3 1^st^ day | 1094.935 | 1.962 _1, 9_ | 0.195 |
